# Matrix Metalloproteinase-9 and Postoperative Outcomes in Carotid Endarterectomy: A Systematic Review

**DOI:** 10.3390/jcm14093235

**Published:** 2025-05-07

**Authors:** João Gonçalves-Silva, Mariana Fragão-Marques, Hugo Ribeiro, Susana I. Sá, João Rocha-Neves

**Affiliations:** 1 Faculty of Medicine, University of Porto, 4200-319 Porto, Portugal; marianaif.rm@gmail.com; 2 RISE-Health, Faculty of Medicine, University of Porto, 4200-319 Porto, Portugal; sasusana@med.up.pt; 3 Community Palliative Care Support Team Gaia, Health Local Unit Gaia and Espinho, 4434-502 Vila Nova de Gaia, Portugal; hribeiroff@gmail.com; 4 Faculty of Medicine, University of Coimbra, 3000-370 Coimbra, Portugal; 5 Coimbra Institute for Biomedical Research, 3000-548 Coimbra, Portugal; 6 Departamento de Medicina da Comunidade, Informação e Decisão em Saúde, Faculty of Medicine, University of Porto, 4200-319 Porto, Portugal; 7 Unit of Anatomy, Department of Biomedicine, Faculty of Medicine, University of Porto, 4200-319 Porto, Portugal

**Keywords:** vascular remodeling, proteolytic enzymes, postoperative recovery, carotid stenosis, extracellular matrix proteins, gelatinase B

## Abstract

**Background/Objectives:** Carotid endarterectomy (CEA) is the treatment of choice for severe symptomatic and asymptomatic carotid artery stenosis. Nonetheless, it carries risks and several complications, including stroke and death. Previous studies have indicated that elevated matrix metalloproteinase-9 (MMP-9) levels may serve as biomarkers for adverse outcomes after CEA. This systematic review investigates the association between plasma MMP-9 levels and adverse cardiovascular outcomes following CEA. **Methods:** PubMed/MedLine, Scopus and Web of Science were searched for studies assessing the relationship between plasma MMP-9 levels and postoperative outcomes after CEA. Assessment of studies’ quality was performed using the National Heart, Lung, and Blood Institute (NHLBI) Study Quality Assessment Tool for observational cohorts and cross-sectional studies. **Results:** Five studies were included (*n* = 891 participants). All five were retrospective cohort studies. MMP-9 was significantly higher in patients who presented with a combination of amaurosis fugax, central retinal artery occlusion, TIA and minor/major stroke at follow-up. However, individual outcomes like TIA or stroke did not consistently correlate with MMP-9 levels. Additionally, increased MMP-9 levels were also associated with cognitive dysfunction post CEA. **Conclusions:** Despite the potential of MMP-9 levels to serve as a biomarker for predicting postoperative cerebrovascular complications, this review presents limitations, including a high risk of bias in included studies and variability in methodologies. There is a need for further research with larger cohorts to validate these findings and improve risk stratification and management strategies for patients undergoing CEA.

## 1. Introduction

Carotid endarterectomy (CEA) is indicated as the preferred treatment for severe (≥70%) symptomatic and asymptomatic carotid artery stenosis (CAS) [1]. Although surgical intervention with CEA may reduce the risk of stroke and other significant outcomes in patients with CAS, it is associated with a potential risk of complications during follow-up. A randomized trial observed that the frequency of a major stroke or death at 3-year follow-up after CEA is up to 14.9% [2]. In another study involving 3061 patients undergoing CEA over a 10-year period, the combined incidence of stroke, myocardial infarction or death was reported to reach as high as 7.4% among high-risk patients [3]. These outcomes highlight the importance of identifying biomarkers that could assess the risk of major complications after the procedure.

Matrix metalloproteinases (MMPs) are a family of zinc-dependent endopeptidases responsible for the degradation and remodeling of the extracellular matrix (ECM) and other non-ECM substrates [4,5,6]. They are secreted as inactive proenzymes by various cell types (including inflammatory cells and fibroblasts) and become active through limited proteolysis [4,5,6]. It was suggested that MMPs contribute to vascular remodeling and the destabilization of atherosclerotic plaques in the internal carotid artery [4]. It was proposed that higher plasma MMP-9 levels may serve as markers of brain ischemia in CAS patients [5,6]. It has also been reported that patients exhibiting spontaneous cerebral embolization presented significantly elevated plasma levels of MMP-9 (92 kD, gelatinase B) [7]. Emerging evidence suggests that elevated preoperative MMP-9 levels may indeed indicate higher ischemic risk in asymptomatic carotid stenosis. Studies demonstrate that MMP-9 expression correlates with plaque instability and cerebrovascular events, with potential implications for preoperative risk stratification [8]. Thus, measuring MMP-9 levels could be useful in predicting clinically relevant complications after CEA.

Given the present evidence gap, this study aimed to systematically review the literature for studies investigating the association of MMP-9 levels with the incidence of major adverse cardiovascular outcomes after CEA.

## 2. Materials and Methods

This systematic review was conducted according to the Preferred Reporting Items for a Systematic Review and Meta-Analysis (PRISMA) statement and the AMSTAR-2 critical appraisal tool [9,10]. An institutional review board’s ethical approval was not obtained due to the nature of this study. The review protocol has been registered at Prospero (reference: CRD42024583801).

### 2.1. Search Strategy

A systematic search was performed in three databases–Pubmed/MEDLINE, Scopus and Web of Science–on 29 August 2024. The keywords used and the queries investigated are demonstrated on Supplementary Table S1. The reference lists of the gathered reports were manually searched for additional relevant studies.

### 2.2. Selection Criteria

Inclusion criteria consisted of all original articles in which the association between MMP-9 levels and outcomes after CEA was assessed in humans. No exclusion criteria based on the publication language or date were applied. Systematic reviews and studies with populations under 20 patients were excluded. Other exclusion criteria included animal studies, pediatric populations (<18 years old), patients undergoing non-CEA surgeries (including carotid artery stenting), studies focusing solely on pre-surgical rather than post-surgical outcomes and the absence of a full text.

### 2.3. Study Selection and Data Extraction

After duplicates were removed, two authors (J.G.-S. and J.R.-N.) independently participated in study selection; any disagreement was resolved by a third author (M.F.-M.). First, studies were selected by title and abstract, and the remaining ones were eligible for full-text assessment. Efforts were made to contact the authors to obtain the full, publicly available texts. The selected studies were carefully reviewed to avoid repeated populations.

Two authors (J.G.-S. and J.R.-N.) independently extracted data from included studies. Data were extracted on the year of publication, country, center of recruitment, study design, recruitment time, number of participants undergoing CEA, participants’ age, gender distribution, frequency of cardiovascular comorbidities and carotid symptomatic status. The primary outcomes analyzed were a collection of significant cardiovascular events that occurred after carotid endarterectomy (CEA). These included long-term occurrences of acute myocardial infarction (AMI), amaurosis fugax, transient ischemic attack (TIA), stroke, central retinal artery occlusion and all-cause mortality. Additionally, acute cognitive dysfunction following CEA was evaluated as a secondary outcome.

### 2.4. Assessment of Study Quality

Concerning qualitative assessment, the National Heart, Lung, and Blood Institute (NHLBI) Study Quality Assessment Tool was used for observational cohort and cross-sectional studies (accessed on 3 January 2025; available at https://www.nhlbi.nih.gov/health-topics/study-quality-assessment-tools). This assessment was independently performed by two authors (J.G.-S. and J.R.-N.), and when disagreements were observed, decisions were made by mutual consensus after a third-party review (M.F.-M.). As there were no randomized controlled trials among the articles that were included, the use of a specific scale for RCTs was not necessary.

The quality of evidence for the included articles was also evaluated using the Grading of Recommendations, Assessment, Development and Evaluation (GRADE) approach. Articles were classified into four levels of Quality of Evidence (high, moderate, low and very low) [10].

## 3. Results

### 3.1. Search Results

**Figure 1:**
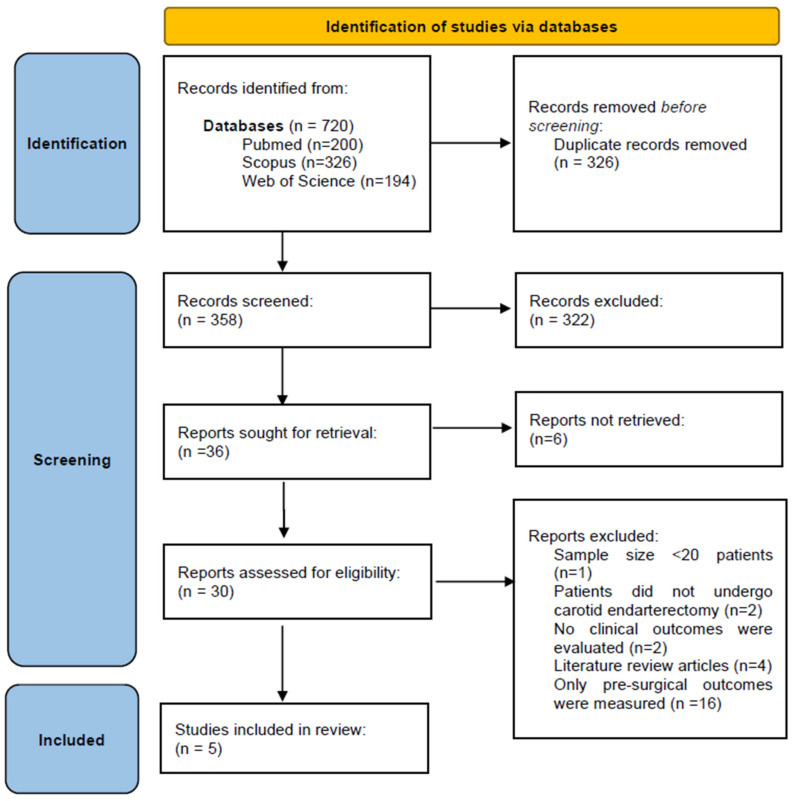
Flow diagram according to PRISMA statement regarding the process of identification and selection of the studies [8].

Following removal of duplicate articles, 358 studies were identified for screening. After evaluating titles and abstracts, 322 studies were excluded. Therefore, 36 studies were selected for full-text assessment. During this phase, twenty-five studies were excluded and six could not be retrieved even after contacting the respective authors (Figure 1). The studies excluded upon full-text assessment were as follows: studies with a sample size of fewer than 20 patients (*n* = 1), studies evaluating patients who had not undergone CEA (*n* = 2), studies that did not assess clinical outcomes (*n* = 2), literature review articles (*n* = 4) and studies focusing solely on pre-surgical rather than post-surgical outcomes (*n* = 16). Thus, five articles were included in the systematic review.

### 3.2. Description of Studies

From the included studies in this systematic review, four articles were retrospective observational cohort studies and one article was a prospective observational cohort study. These were performed in four different countries within two continents: two were from North America [11,12] and three from Europe [13,14,15]. A total of 891 patients were assessed, with a minimum of 23 and a maximum of 543 patients per study (Table 1). The mean participants’ age ranged between 67.5 and 73.9 years old, while the percentage of male participants varied between 36.5% and 71.6%. Symptomatic carotid disease was reported in three out of the five studies and had a prevalence between 12.3% and 81.4%. The demographics and comorbidities of the populations included in the studies are presented in Table 2.

### 3.3. Study Quality

**Figure 2:**
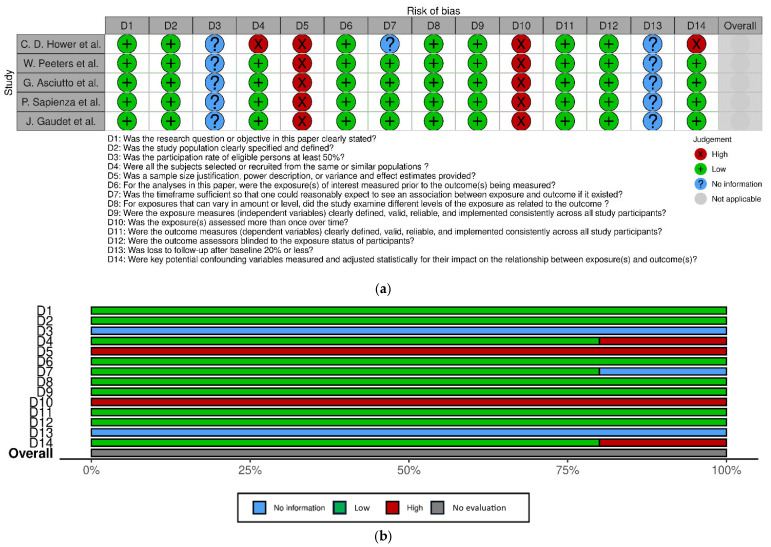
(**a**) Risk of bias of all observational studies included in the systematic review, displayed by article [11,12,13,14,15]. (**b**) Risk of bias of all included observational studies, displayed by item.

The risk of bias for each selected observational cohort is individually displayed in Figure 2a. The overall judgment per evaluated quality item regarding the included studies is shown in Figure 2b. The GRADE evaluation is displayed on Table 1.

According to the National Heart, Lung, and Blood Institute (NHLBI) Study Quality Assessment Tool (Figure 2a,b), the items most frequently associated with a high risk of bias included sample size justification, power description, variance and effect estimates (D5) and exposure assessment (D10), items that were not adequately addressed in any of the included studies [11,12,13,14,16].

Also, all five studies were classified as having a low grade of evidence according to the GRADE evaluation [16], meaning that the true effect might be markedly different from the estimated effect in those studies.

### 3.4. Main Findings

Follow-up time after CEA ranged between a median of 2.4 years and 74 months (Table 3). MMP-9 was significantly higher in patients who presented with a combination of amaurosis fugax, central retinal artery occlusion, TIA and minor/major stroke at follow-up [13]. However, when considering the individual outcomes of TIA or stroke in another study [14], neither endpoint presented an association with MMP-9 levels. Similarly, when an included research paper [14] evaluated the composite outcome of death of vascular origin, non-fatal stroke, non-fatal myocardial infarction and any arterial vascular intervention that had not already been planned at the time of inclusion, there was no association of MMP-9 levels with the endpoint. Conversely, MMP-9 was significantly higher in patients with cognitive dysfunction after CEA [11]. Additionally, in another study, pro-MMP-9 was significantly lower in patients who presented with a composite endpoint of amaurosis fugax, TIA and stroke [12]. All these results are presented in Table 4.

## 4. Discussion

This systematic review aimed to investigate the relationship between MMP-9 levels and clinical outcomes following CEA.

A total of five observational cohort studies were included, with a total of 891 participants being evaluated. It was found that elevated MMP-9 levels were significantly associated with a composite endpoint of amaurosis fugax, TIA, stroke and central retinal artery occlusion in one of the studies included [13]. However, inconsistencies were found concerning individual outcomes like TIA or stroke, which may reflect the multifactorial nature of those events post CEA, whose presence may not be exclusively dependent on MMP-9 levels [14]. A plausible explanation for the contradictory findings reported by Asciutto et al. may be that such events are not directly related to MMP-9 levels and their inflammatory activity within the extracellular matrix. Additionally, these findings may indicate that, within composite outcomes, the impact of stroke and TIA might be overestimated, as they may carry less clinical or biological significance but are interpreted alongside more substantial components of the composite endpoint. Additionally, increased MMP-9 levels were linked to cognitive dysfunction post surgery [11]. In terms of qualitative assessment, we concluded that the included studies had a high risk of bias, mainly due to issues related to sample size justifications and the exposure assessment, which may affect the reliability of the results.

Those findings are consistent with the currently available research regarding the pathophysiology of carotid artery disease and its complications. Previous studies have demonstrated a correlation between elevated MMP-9 levels and increased cardiovascular events, suggesting that MMP-9 levels could be potentially used as a biomarker for adverse cardiovascular outcomes [17,18]. Also, MMP-9 is part of the pathophysiology of atherosclerotic disease, mainly because of its role in ECM remodeling and inflammation [4,19,20]. It was equally shown that elevated MMP-9 levels contribute to plaque destabilization and rupture, which are critical events in the origin of cardiovascular outcomes [21,22]. By degrading ECM components, MMP-9 enables the infiltration of inflammatory cells into atherosclerotic plaques, worsening local inflammation and promoting plaque instability [20]. In fact, there is recent evidence that MMP-9 is one potential biomarker for distinguishing between stable vs. unstable atherosclerotic plaque formation [23]. Additionally, MMP-9 has the ability of activating various cytokines and chemokines, further enhancing inflammatory responses within the vascular wall [24]. That being said, MMP-9 inhibitors were suggested as a possible therapeutic strategy to stabilize plaques and reduce cardiovascular events [25]. Consequently, MMP-9 has the potential to work not only as a marker of plaque vulnerability but also as a potential target for the treatment and management of atherosclerosis and its complications.

In fact, reliable biomarkers that can predict immediate surgical outcomes and long-term neurological and cognitive deficits are needed, reinforcing the potential relevance of MMP-9 in this context. The stratification and measurement of MMP-9 levels may be clinically significant for managing patients undergoing CEA. In fact, if we identify patients with elevated MMP-9 levels, we could enhance risk stratifications, which would allow clinicians to better adapt postoperative monitoring of those patients and make intervention strategies more effective [26]. Knowing that, patients with higher MMP-9 levels might benefit from more intensive follow-up protocols to mitigate the risk of adverse outcomes such as stroke or cognitive decline [11,13]. Additionally, by having a deeper understanding of the dynamics of MMP-9 and its relation to clinical outcomes, we could enhance the development of targeted therapies aimed at modulating its activity, potentially improving patients’ prognosis after CEA.

This systematic review has several strengths, including a comprehensive search strategy across multiple databases and adherence to established reporting guidelines (PRISMA) [9]. Also, including diverse populations from different geographical regions enhances the generalizability of the findings.

However, our study has some limitations. The high risk of bias across included studies raises concerns about the reliability of the results. The retrospective nature of the majority of these studies limits causal inferences. Additionally, few articles were eligible for this systematic review, and the majority of those had a small sample size without sample justifications and power descriptions, leading to low precision of obtained results. Moreover, there was significant heterogeneity amongst studies regarding most baseline patient characteristics, study designs, methodology and outcome definitions, which might have contributed to inconsistencies in findings. The low number of included studies and the extensive differences between primary studies determined the impossibility of performing a meta-analysis and restricted the ability to draw definitive conclusions regarding the relationship between MMP-9 levels and clinical outcomes after CEA. Nonetheless, to enable more robust and reliable conclusions, further studies in this area are warranted. Future research should aim to standardize MMP-9 measurement techniques to establish clinically meaningful cut-off values, adopt consistent outcome definitions in line with reporting guidelines, rigorously control for relevant confounding variables, ensure adequate sample sizes supported by power calculations and explore potential therapeutic strategies targeting MMP-9.

## 5. Conclusions

MMP-9 levels were significantly higher in patients with cerebrovascular endpoints after CEA. However, inferring results should be cautiously interpreted due to the limitations in available evidence. This systematic review suggests a need for additional large cohorts with the primary aim of assessing the effect of MMP-9 levels on the incidence of cerebro- and cardiovascular outcomes at follow-up in patients undergoing CEA in order to validate the current findings.

## Figures and Tables

**Table 1:** Identification and summary description of the selected studies from where data were retrieved.

Study (Year)	Study Design	Study Center	Recruitment Time	Sample Size (Patients)	GRADE Evaluation	No. CEA
Hower et al. [12] (2000)	Retrospective Cohort study	Multicenter (Medical College of Wisconsin and the Clement J. Zablocki Veterans Affairs Medical Center, Milwaukee, Wisconsin)	NA	23	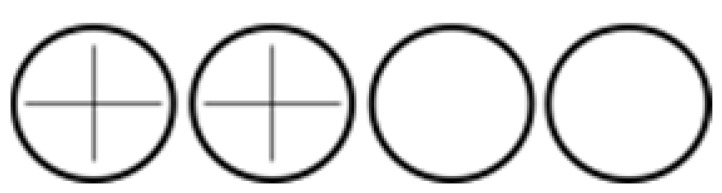 Low	23
Peeters et al. [15] (2011)	Retrospective Cohort study	Multicenter (St. Antonius Hospital Nieuwegein and University Medical Center Utrecht)	March 2002 and February 2006	543	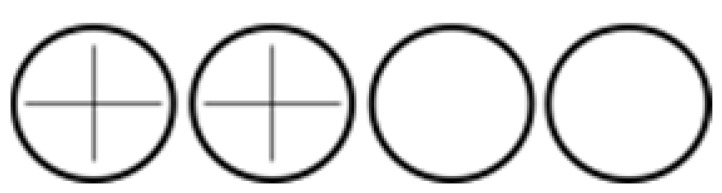 Low	543
Asciutto et al. [14] (2015)	Retrospective Cohort study	Vascular Centre Malmö-Lund, Skåne University Hospital, Malmö, Sweden	October 2005 and October 2009	209	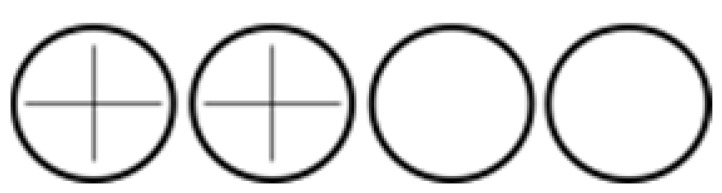 Low	221
Sapienza et al. [13] (2009)	Retrospective Cohort study	Department of Surgery “Pietro Valdoni”, University of Rome “La Sapienza”	January 1999 and December 2003	52	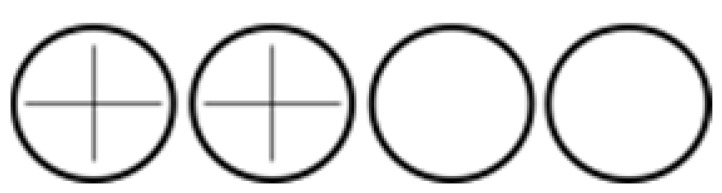 Low	621
Gaudet et al. [11] (2010)	Prospective Cohort study	New York Presbyterian Hospital, Columbia University	2005–2007	64	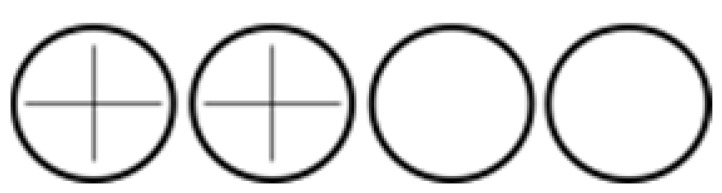 Low	73

CEA—carotid endarterectomy. The “++” symbol means “Low evidence according to GRADE evaluation”. NA—not available.

**Table 2:** Demographics and comorbidities of the population samples for each study.

Study (Year)	Mean Age (Years)	Male *n* (%)	Arterial Hypertension *n* (%)	Dislipidemia *n* (%)	Diabetes Mellitus *n* (%)	Smoking History *n* (%)	Coronary Artery Disease *n* (%)	Symptomatic Carotid Disease *n* (%)
Hower et al. [12] (2000)	73.0	13 (56.5%)	NA	NA	NA	NA	NA	NA
Peeters et al. [15] (2011)	67.5	389 (71.6%)	351 (65.0%)	322 (59.4%)	99 (18.3%)	141 (26.7%)	115 (21.3%)	442 (81.4%)
Asciutto et al. [14] (2015)	69.7	NA	NA	NA	NA	NA	NA	NA
Sapienza et al. [13] (2009)	73.9	19 (36.5%)	29 (55.8%)	NA	13 (25.0%)	26 (50.0%)	18 (34.6%)	23 (44.2%)
Gaudet et al. [11] (2010)	NA	47 (64.4%)	50 (68.5%)	53 (72.6%)	11 (15.1%)	46 (63.0%)	NA	9 (12.3%)

NA—not available.

**Table 3:** Outcomes after CEA.

	Acute Cognitive Dysfunction *n* (%)	Acute Cognitive Dysfunction Definition	Follow-Up, Median (Range)	Long-Term Acute Myocardial Infarction, *n* (%)	Long-Term Amaurosis Fugax, *n* (%)	Long-Term Transient Ischemic Attack, *n* (%)	Long-Term Stroke *n* (%)	Long-Term Central Retinal Occlusion, *n* (%)	Long-Term All-Cause Mortality, *n* (%)
Hower et al. [12] (2000)	NA	NA	6 weeks	NA	4 (17.4%)	2 (8.7%)	1 (4.3%)	NA	NA
Peeters et al. [15] (2011)	NA	NA	2.4 (0.97–3.0) years	34 (6.3%)	NA	NA	31 (5.7%)	NA	22 (4.1%)
Asciutto et al. [14] (2015)	NA	NA	39.6 (23.0–56.2) months	NA	NA	5 (2.3%)	12 (5.4%)	NA	NA
Sapienza et al. [13] (2009)	NA	NA	74.0 (65.0–83.0) months	NA	6 (11.5%)	12 (23.1%)	1 (1.9%)	1 (1.9%)	NA
Gaudet et al. [11] (2010)	12 (16.4%)	Patients with an average z-score of ≤−1.5 using 6 neurological tests for the assessment of cognitive dysfunction.	NA	NA	NA	NA	NA	NA	NA

NA—not available.

**Table 4:** MMP-9 levels according to outcome at follow-up and quantification method.

Study (Year)	Outcome/Symptomatic Definition	MMP-9 Levels/Activity in Asymptomatic vs. Symptomatic Patients. (*p* Value)	MMP-9–Measurement Kit/Sample
Hower et al. [12] (2000)	Composite of endpoints: amaurosis fugax, TIAs and stroke.	Pro-MMP-9 levels: 17.42 (14.28–20.56) ng vs. 8.21 (5.86–10.56) ng; (*p* < 0.05)	ELISA kit (Biotrak assays) from Amersham Life Sciences (Buckinghamshire, England). Intraoperative-atheroscleortic plaque
Peeters et al. [15] (2011)	Composite of endpoints: any death of vascular origin, non-fatal stroke, non-fatal myocardial infarction and any arterial vascular intervention that had not already been planned at the time of inclusion.	0.76 (0.40–1.42) AU vs. 0.95 (0.46–1.54) AU; (*p* = 0.14)	Biotrak activity assays (MMP-9 RPN-2634) from GE Healthcare Life-Sciences, Buckinghamshire, UK. Intraoperative-atheroscleortic plaque
Asciutto et al. [14] (2015)	Stroke; TIA.	Stroke: 921.36 (± 1247.18) AU/g vs. 1477.80 ± (2554.18) AU/g; (*p* = 0.683) TIA: 968.04 ± 1364.9 AU/g vs. 352.47 ± 162.5 AU/g; (*p* = 0.491)	MMP kit from Mesoscale (Gaithersburg, MD, USA), following the manufacturer’s instructions. Intraoperative-atheroscleortic plaque
Sapienza et al. [13] (2009)	Composite of endpoints: TIA, amaurosis fugax, central retinal artery occlusion and minor or major stroke.	Early symptoms (6 months to 3 years after CEA): 11 (8–14) ng/mL vs. 40 (34–46) ng/mL; (*p* < 0.0001) Late symptoms (over 3 years after CEA): 24 (16–32) ng/mL vs. 87 (77–97) ng/mL; (*p* < 0.0001)	ELISA technique provided by Amersham Pharmacia. Preoperative blood sample
Gaudet et al. [11] (2010)	Patients with an average z-score of ≤−1.5 on an assessment using six neurological tests representing a range of cognitive domains were considered to have cognitive disfunction.	43.18 (38.74–47.62) ng/mL vs. 81.66 (69.41–91.91); (*p* < 0.005)	ELISA kit from R&D Systems (Minneapolis, MN, USA). Preoperative blood sample.

TIA—transient ischemic attack; MMP-9—matrix metalloproteinase-9.

## Supplementary Materials

The following supporting information can be downloaded at: https://www.mdpi.com/article/10.3390/jcm14093235/s1, Table S1: Keywords and queries investigated in the systematic review.
