# Supplementary material for: Matrix Metalloproteinase-9 and Postoperative Outcomes in Carotid Endarterectomy: A Systematic Review
Source: J Clin Med. 2025 May 7;14(9):3235. doi: 10.3390/jcm14093235 (PMC12072748; doi:10.3390/jcm14093235)
Supplement: Supplementary file 1 [file jcm-14-03235-s001.zip › jcm-3586330-supplementary.pdf]

Supplementary Table S1. Keywords and queries investigated in the systematic review.

Bibliographic source	Search term	No of reports 29/Aug/2024
**Pubmed/MEDLINE**	((“matrix metalloproteinases”) OR (“MMPs”) OR (“matrix metalloproteinase 9”) OR (“MMP-9”) OR (“MMP9”) OR (“Gelatinase B”)) AND ("Endarterectomy" OR "Endarterectomy, Carotid" OR “carotid endarterectomy” OR “Carotid Endarterectomies”)	200
**Scopus**	((“matrix metalloproteinases”) OR (“MMPs”) OR (“matrix metalloproteinase 9”) OR (“MMP-9”) OR (“MMP9”) OR (“Gelatinase B”)) AND ("Endarterectomy" OR "Endarterectomy, Carotid" OR “carotid endarterectomy” OR “Carotid Endarterectomies”)	326
**Web of science**	((“matrix metalloproteinases”) OR (“MMPs”) OR (“matrix metalloproteinase 9”) OR (“MMP-9”) OR (“MMP9”) OR (“Gelatinase B”)) AND ("Endarterectomy" OR "Endarterectomy, Carotid" OR “carotid endarterectomy” OR “Carotid Endarterectomies”)	194
